# The influence of hydrogen peroxide and histamine on lung permeability and translocation of iridium nanoparticles in the isolated perfused rat lung

**DOI:** 10.1186/1743-8977-2-3

**Published:** 2005-06-27

**Authors:** James J Meiring, Paul JA Borm, Karim Bagate, Manuela Semmler, Jürgen Seitz, Shinji Takenaka, Wolfgang G Kreyling

**Affiliations:** 1 Particle Research Core, Institute für Umweltmedizinische Forschung (IUF) an der Heinrich-Heine Universität gGmbH, Auf'm Hennekamp 50 D-40225 Düsseldorf, Germany; 2 GSF Forschungszentrum für Umwelt und Gesundheit, Ingolstädter Landstr. 1, Institute for Inhalation Biology & Focus Network Aerosols and Health, D-85746 Neuherberg / München, Germany; 3 Centre of Expertise in Life Sciences (CEL), Zuyd University, PO Box 550, 6400 AN HEERLEN, The Netherlands

**Keywords:** endothelium, translocation, ultrafine particles, isolated perfused lung, permeability.

## Abstract

**Background:**

Translocation of ultrafine particles (UFP) into the blood that returns from the lungs to the heart has been forwarded as a mechanism for particle-induced cardiovascular effects. The objective of this study was to evaluate the role of the endothelial barrier in the translocation of inhaled UFP from the lung into circulation.

**Methods:**

The isolated perfused rat lung (IPRL) was used under negative pressure ventilation, and radioactive iridium particles (18 nm, CMD, ^192^Ir-UFP) were inhaled during 60 minutes to achieve a lung burden of 100 – 200 μg. Particle inhalation was done under following treatments: i) control perfusion, ii) histamine (1 μM in perfusate, iii) luminal histamine instillation (1 mM), and iv) luminal instillation of H_2_O_2_. Particle translocation to the perfusate was assessed by the radioactivity of ^192^Ir isotope. Lung permeability by the use of Tc^99m^-labeled diethylene triamine pentaacetic acid (DTPA). In addition to light microscopic morphological evaluation of fixed lungs, alkaline phosphatase (AKP) and angiotensin converting enzyme (ACE) in perfusate were measured to assess epithelial and endothelial integrity.

**Results:**

Particle distribution in the lung was homogenous and similar to in vivo conditions. No translocation of Ir particles at negative pressure inhalation was detected in control IPL, but lungs pretreated with histamine (1 μM) in the perfusate or with luminal H_2_O_2 _(0.5 mM) showed small amounts of radioactivity (2–3 % dose) in the single pass perfusate starting at 60 min of perfusion. Although the kinetics of particle translocation were different from permeability for ^99m^Tc-DTPA, the pretreatments (H_2_O_2_, vascular histamine) caused similar changes in the translocation of particles and soluble mediator. Increased translocation through epithelium and endothelium with a lag time of one hour occurred in the absence of epithelial and endothelial damage.

**Conclusion:**

Permeability of the lung barrier to UFP or nanoparticles is controlled both at the epithelial and endothelial level. Conditions that affect this barrier function such as inflammation may affect translocation of NP.

## Introduction

Epidemiological studies have demonstrated an increased morbidity and mortality by particulate air pollution [1,2]. The highest relative risk for mortality and hospital admissions were observed in subjects with existing pulmonary disease including asthma and COPD [1,2]. The exact mechanism by which PM can adversely affect humans remains unknown, but several hypotheses have been forwarded. These include that PM causes pulmonary inflammation causing release of factors that influence blood coagulation [3], reduced lung function [4], increased blood plasma viscosity [5], reduced heart rate variability [6,7] and destabilisation of atheromatous plagues [8]. Some of these effects are attributed to translocated nanoparticles based on their potential effects on vascular function [9,10], blood coagulation [11], mitochondrial function [12] and Ca-flow [13,14].

Nanoparticles have been shown to translocate from lung to the circulation [15-18], but most of the inhaled dose remains in the lung interstitium [19] even up to several years [20]. Therefore it seems that not the epithelial but the endothelial barrier is more important in prevention of translocation to the blood. Enhanced lung permeability has been measured by increased Clara-cell protein in blood [21] or enhanced DTPA clearance in the lung [22] after ozone and hyperoxia. Recent work in rabbit isolated perfused lungs shows that nanoparticles themselves can influence microvascular permeability measured by weight gain after occlusion [23]. However, since the mechanisms of nanoparticle transport on a sub-cellular level are unknown it remains to be determined whether the above indices of lung permeability are related to translocation of nanoparticles. Particles may also cause the release of vasoactive mediators such as histamine, which was shown to be increased in plasma of hamster after instillation of diesel exhaust particles [24]. Histamine is well known to induce vascular permeability through its action on endothelial H_1_-receptor [25]. Finally, by oxidative stress mechanisms ambient and nanoparticles can cause activation of lung alveolar macrophages and epithelial cells that result in the production of pro-inflammatory cytokines such as TNF and Il-1 in humans [26] and rat models [27] that are typically associated with increased lung permeability [28,29].

The objective of this study was to assess nanoparticle translocation in relation to permeability changes for small molecules and integrity of epithelial and endothelial monolayers. In order to manipulate permeability in the absence of neutrophil recruitment and activation we used an isolated perfused rat lung. Several treatments to modify lung permeability in-vitro were applied including oxidative stress by instillation of hydrogen peroxide and endothelial permeability by histamine in the perfusate. These treatments were selected for their relevance to conditions of patients with pulmonary or systemic complications. Particle translocation was assessed by the inherent radioactivity of 18 nm size iridium nanoparticles (^192^Ir-UFP).

## Materials & methods

### Animals and surgical procedure

Adult, healthy, male Wistar-Kyoto rats (WKY/Kyo@Rj rats, Janvier, France) (200–250 g) were housed in pairs in a humidity (55% relative humidity) and temperature (22°C) controlled room. They were maintained on a 12-h day/night cycle. Rats were allowed to acclimate to the facility for a minimum of 10 days prior to use. When the experiments were performed rats were more than 17 weeks of age. The studies were conducted under federal guidelines for the use and care of laboratory animals and were approved by the Oberbayern Government and by the GSF Institutional Animal Care and Use Committee. Surgical procedure for lung isolations was done according the method of Uhlig and Wollin [30]. Briefly, rats were anaesthetized intraperitoneally with 80 mg/kg ketamin. Deep anaesthesia was characterized by a lack of response to toe pinching. Heparin (500 IU) was injected via the tail vein. A midline incision was made from the pelvic region to the neck of the rat. With the ventilator operating, the trachea was cannulated using a rigid catheter and the catheter was attached to the ventilator. Therefore lung were ventilated from the start of the whole procedure. The animals were exsanguinated opening the aorta abdominalis after deep intraperitoneal anesthesia with ketamine (100 mg/100 g body weight) and xylazine (0.5 mg/100 g body weight). After anesthesia a longitudinal ventral incision was made to open the thoracic and abdominal cavity and it was held open using clamps. The thymus was removed and the apex of the heart was cut off to introduce a cannula into the pulmonary artery. A slight perfusion flow of around 1 ml/min was maintained before inserting cannula. Care was taken not to introduce any air bubbles into the pulmonary artery. The left atrium was cannulated by advancing the venous cannula through the mitral valve. A ligature was placed around the heart to keep both cannula's in place. The aortic cannula was then attached to the lining fed through the Perspex lid of the 500 mL negative-pressure-chamber. After the Perspex lid was fully mounted on the chamber negative pressure ventilation started. The respiratory settings during the negative pressure ventilation were 65 breaths per min. Regular sighs were introduced (hyperventilation) to improve function of lungs.

### Isolated lung perfusion

The IPL-4401 Isolated lung ventilation perfusion system (FMI GmbH Oberbach) was used for our study. Additional negative pressure chamber has been constructed by GSF -National Research Centre for Environment and Health. The system in brief consists of a small animal ventilator, jacketed upper media reservoir, negative-pressure-chamber holding the heart-lung-bloc, perfusion lines, peristaltic pump and pulmonary artery pressure transducer. The computer software (FMI GmbH Oberbach) was operated by a 386 personal computer and allowed constant monitoring of pulmonary blood pressure. The upper media reservoir, ventilation chamber and perfusion lines were held at 37°C by a re-circulating water-bath. The perfusion medium was selected based on its extensive use in isolated organ perfusion and consisted of a modified Krebs-Ringer -bicarbonate buffer. Krebs-Ringer composition was as follows (mM): NaCl 118 ; KCl 5.9 ; CaCl_2 _2.5 ; MgSO_4 _1.2 ; NaH_2_PO_4 _1.2 ; NaHCO_3 _24.9 ; glucose 11.1, pH was adjusted at 7.4. It was then mixed at a ratio 1:1 with Haemacell solution (Hoechst Marion Roussel). The buffer was pre-warmed and gassed with 95% O_2 _and 5% CO_2 _at a rate low enough to prevent excessive frothing of the medium. The medium flowing through the system passed through a bubble trap prior to reaching the lungs and the buffer pH was continuously monitored throughout the experiment. Respiratory rate was set at 65 breaths per min. Lungs were inflated at a maximum negative pressure of -1.5 kPa in the chamber. Stroke volume was set at 10 ml to achieve a tidal volume of usually 3–4 ml (because of the altered compliance of the lungs). The lungs were expanded (sighed) every 4 minutes applying a negative pressure of -2.5 kPa to the chamber. Optimal perfusion settings included perfusion rate of 5 ml/min and a medium pH of 7.4. Perfusion pressure was not constant but was kept between 10 and 14 kPa.

### Particle generation and exposure

**Figure 1:**
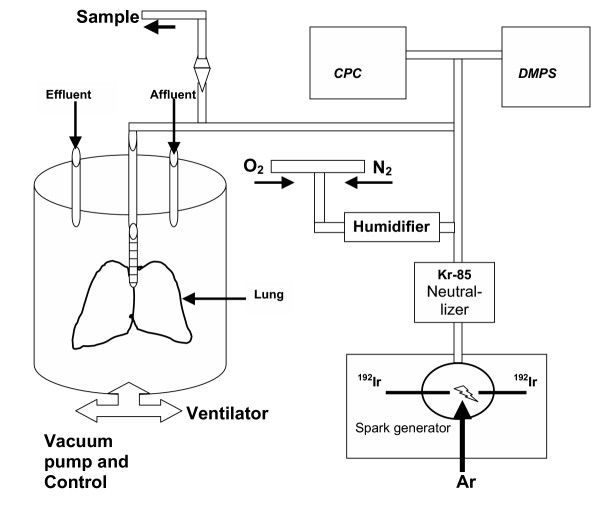
Diagram of the experimental perfusion system used for this study. The ultrafine iridium particles (Ir-UFP) radiolabelled with ^192^Ir were produced in the spark generator. At the exit of spark generator the aerosol was quasi-neutralized by a radioactive ^85^Kr source. The aerosol was diluted with nitrogen and with oxygen and adjusted to obtain 20% oxygen and was air conditioned at 50–60 % relative humidity. The particle size distribution and number concentration were monitored by a differential mobility particle sizer (DMPS) and a condensation particle counter (CPC). ^192^Ir-UFP radioactivity of the aerosol was determined by continuous aerosol sampling of a measured volume and integral radioactivity counting. The lungs were perfused at a perfusion rate of 5 ml/min and a stroke volume of 10 ml. Respiration rate set at 65 breaths per minute. Negative ventilation pressure in chamber was regulated with animal ventilator. Lungs were manually expanded (sighed) every 4 minutes by applying a negative pressure of -2.5 kPa to the chamber.

Aerosols of ultrafine iridium particles (Ir-UFP) radiolabeled with ^192^Ir were produced with a spark generator as described previously [15]. Size distribution and number concentration were monitored continuously by a differential mobility particle sizer (DMPS 3070, TSA instruments) and a condensation particle counter (CPC 3022A, TSI Instruments). The size distribution of the ^192^Ir-UFP was aimed to a count median diameter of 17–20 nm (geometric standard deviation 1.6) at a particle concentration of 10^7 ^cm^-3 ^aiming for a tidal volume of 3–4 cm^3 ^at a frequency of 65 /min. The estimated dose under these conditions is 180 μg/hour. A schematical description of the experimental system is shown in Figure 1

### Experimental design

**Figure 2:**
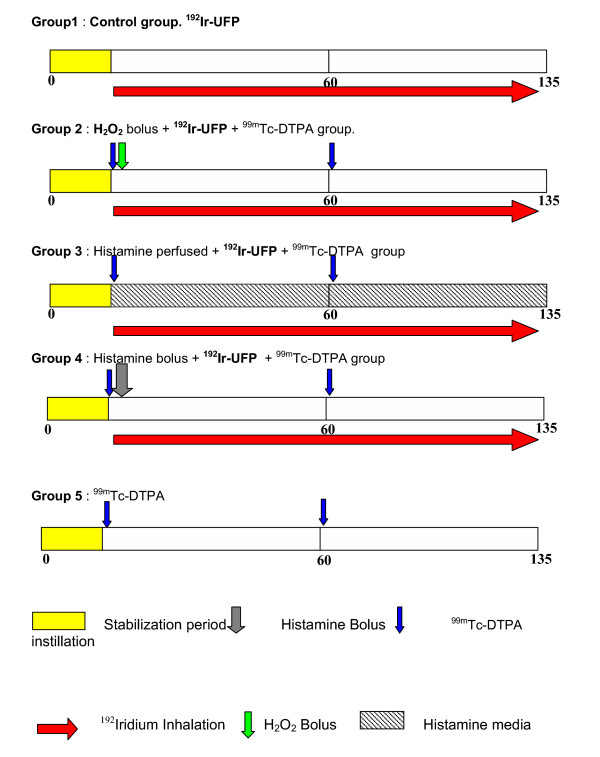
Experimental protocol for isolated perfused rat lungs. All perfusions were done under negative pressure ventilation. Following a 15 minute period of equilibration, during which the lungs were already ventilated and perfused, the experiment started by the inhalation of freshly produced ^192^Ir-UFP from the aerosol line. Intratracheal instillation of ^99m^Tc-DTPA or other instillations were performed at this starting time point. The perfusate was collected continuously and sampled at 15-min time intervals. The following treatments were investigated, *group 1*: control group, only ^192^Ir-UFP inhalation for 120 min; *group 2*: instillation of 50–100 μL ^99m^Tc-DTPA, 500 μl H_2_O_2 _bolus (0.5 mM), ^192^Ir-UFP aerosol inhalation for 120 min; *group 3: *instillation of 50–100 μL ^99m^Tc-DTPA, histamine continuously pefrused during the next 2 hours at concentration 10 μM, ^192^Ir-UFP aerosol inhalation for 120 min; *group 4*: instillation of 50–100 μL ^99m^Tc-DTPA and 500 μl histamine bolus instillation at a concentration of 10 mM, ^192^Ir-UFP aerosol inhalation for 120 min; *group 5*: instillation of 50–100 μL ^99m^Tc-DTPA. For each group 3–4 animals were used.

Following a 15 minute period of equilibration, during which the lungs were already ventilated and perfused, the experiment started by the inhalation of freshly produced ^192^Ir-UFP from the aerosol line. Intratracheal instillation of ^99m^Tc-DTPA or other instillations were performed at this starting time point. The perfusate was collected continuously and sampled at 15-min time intervals (Figure. 2). The following treatments were investigated,

group I: control group, only ^192^Ir-UFP inhalation for 120 min, ;

group II: instillation of 50–100 μL ^99m^Tc-DTPA, 500 μl H_2_O_2 _bolus (10 mM), ^192^Ir-UFP aerosol inhalation for 120 min;

group III: instillation of 50–100 μL ^99m^Tc-DTPA, histamine continuously perused during the next 2 hours at concentration 10 μM, ^192^Ir-UFP aerosol inhalation for 120 min;

group IV: instillation of 50–100 μL ^99m^Tc-DTPA and 500 μl histamine bolus instillation at a concentration of 10 mM, ^192^Ir-UFP aerosol inhalation for 120 min;

group V: instillation of 50–100 μL ^99m^Tc-DTPA.

### Evaluation of ^192^Ir-UFP translocation

The perfusate samples as well as the heart-lung-blocs were analysed for ^192^Ir-UFP activity in a shielded 1-L-well-type gamma-spectrometer. Analysis of ^192^Ir activity was performed in those samples studied for ^99m^Tc-DTPA permeability when the ^99m^Tc activity had decayed – see below. Activity measurements of both isotopes were decay and background corrected. ^192^Ir activity in the perfusate samples were given as a fraction of the total activity found in the perfusate and the heart-lung-bloc.

### Evaluation of lung permeability

Technetium-99^m ^labelled DTPA (^99m^Tc-DTPA; DRN 4362 TechneScan-DTPA, Malinckrodt Medical BV, The Netherlands) was used to evaluate lung permeability. The lyophilised DTPA powder was dissolved in 10 ml sterile ^99m^Tc activity containing saline, which was eluted from the ^99m^Tc generator. The solutions were then allowed to equilibrate for 15 minutes at room temperature. The volume instilled in the trachea was 50–100 μL at a DTPA concentration of 120–250 μg. and a ^99m^Tc activity of 5–10 MBq. The ^99m^Tc-DTPA permeability was studied measuring the activity in the heart-lung-bloc and the perfusate samples. The ^99m^Tc radioactivity was also analysed in the shielded 1-L-well-type gamma-spectrometer at the appropriate photo peak of ^99m^Tc. Since the ^99m^Tc activity was chosen to be at least an order of magnitude higher than the ^192^Ir deposition in the lungs, interference of Compton rays in the ^99m^Tc window originating from ^192^Ir was negligible. Permeated ^99m^Tc activity in the perfusate samples was given as a cumulative fraction of the total instilled activity recovered in the perfusate and the heart-lung-bloc.

### Tissue preparation and microscopy

Immediately after the termination of the lung perfusion the radioactive particles treated lungs were air dried with room air at a pressure 3.5 kPa and subsequent imaging for particle distribution. For histopathology only lungs treated with non-radioactive iridium particles were used. After the experiment, the trachea and pulmonary vein of the IPL were perfused with 2.5 % glutaraldehyde in 0.1 M phosphate buffer (pH 7.2, 340 mOsm) at 25 cm fixative pressure. Post-fixation of the lungs was done by immersion of the whole lung in the same fixation solution for 2 hours at room temperature. t 25 cm fixative pressure. Two slices from left- and right caudal lobes of each animal were embedded in paraffin and 5 μm thick sections were stained with hematoxylin and eosin. Small portions of the left lobe of a a sub-group of 7 animals were embedded in Epon ^®^, and semithin sections (1 μm) were stained by toluidine blue.

### Biochemical analysis of the perfusate

Perfusate samples were analysed for histamine with an ELISA kit from IBL-Hamburg (reference no. RE 59221). The detection limit of the kit was 0,3 ng/ml when using plasma. Angiotensin converting enzyme (ACE) was measured according the kinetic method of Maguire and Price [31] using standards from Bühlmann Laboratories AG, Switzerland (Reference KK-ACK), Protein determination was done according the Bicinchonic acid (BCA) protein assay [32]. Clara-cell protein was measured in perfusate using a sensitive latex immunoassay [21] with a detection limit 1 μg/l perfusate. Alkaline Phosphatase (ALP) determination was done with KIT manufactured by Diasys Diagnostics GmbH Germany Cat no: 104019990314. Samples measured (Beckman DU 640 spectrophotometer) at 25°C at wavelength 405 nm. The increase of the extinction was measured each minute for 3 min and enzyme activity was measured as the difference in extinction divided by the minutes multiply a constant factor 2754 [33].

### Statistical Analysis

Results are expressed as means ± SD, and/or as individual experiments (Fig 5). Differences between treatments were tested for statistical significance by Mann Whitney-test. A value of P < 0.05 was considered significant. All statistics were run with SPSS for Windows XP.

## Results

### Particle distribution and deposition

**Figure 3:**
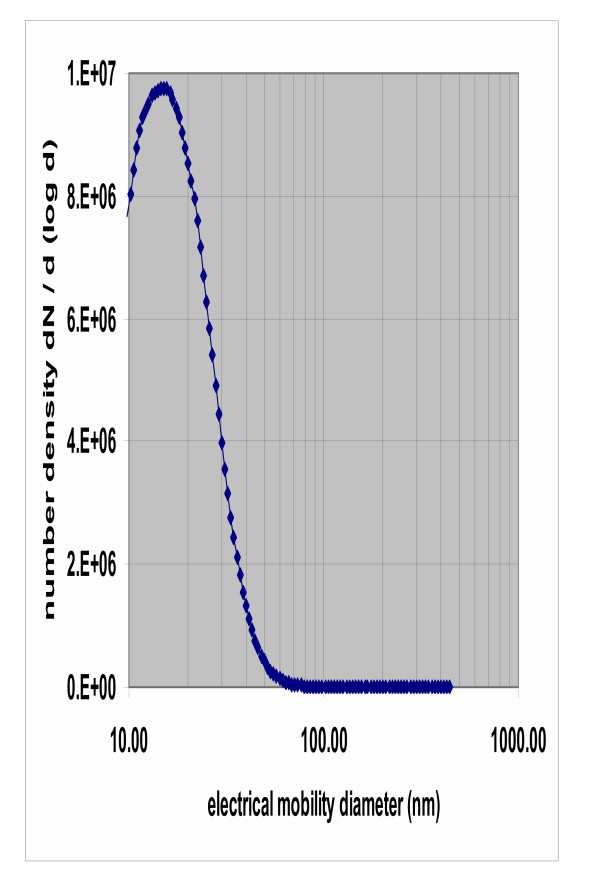
Average particle size distribution in a typical perfusion experiment over entire exposure time (120 min), revealing an average size distribution of count median diameter of16.9 nm, GSD of1.6, aerosol concentration 4.45 10^6 ^cm^-3^, SD 0.13 10^6 ^cm^-3 ^The example shown is from inhalation during histamine perfusion, as shown in Fig 3 (lower panel).

The first set of experiments measured the distribution of ^192^Ir -UFP related radioactivity in the lungs. Deterioration of lung performance was noted based on increasing frequency of inflation to maintain tidal volume, but could not be quantified during negative pressure perfusion in the experimental set-up due to maintain a closed system for radiation protection safety reasons. In a positive pressure using the same equipment, tidal volume, respiration pressure and weight did not change over a 2-hour perfusion period. The particle size distribution in the inhaled aerosol was well reproducible and the count median diameter (CMD) ranged between 16 and 18 nm of particle diameter (Fig 3). Geometric standard deviation (GSD) always was 1.6. Deposition of particles in isolated perfused lungs was compared to animals exposed parallel to the same aerosol and showed similar homogenous distribution, with somewhat lower deposition (data not shown).

### Translocation of ultrafine particles after modified permeability

**Figure 4:**
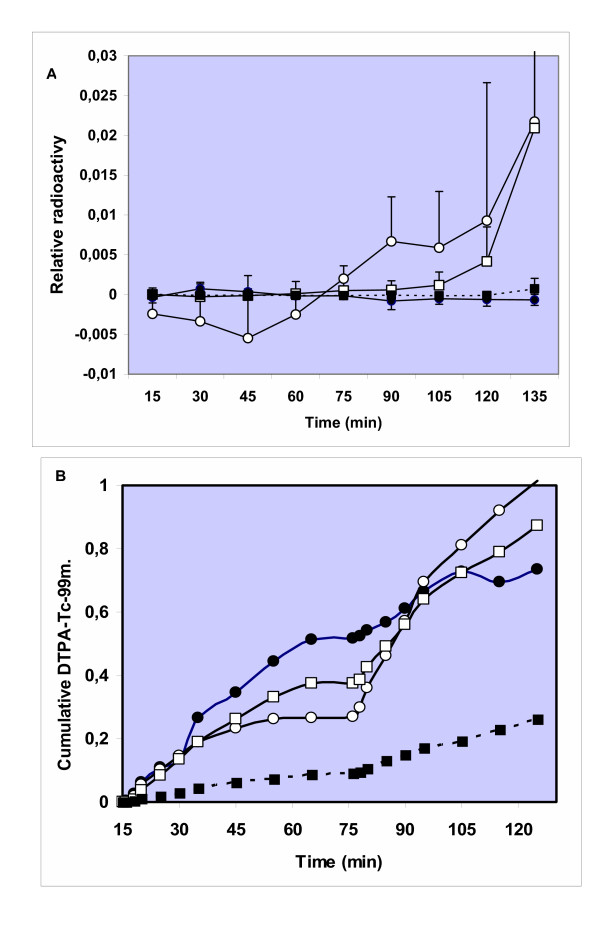
Translocation of ^192^Ir-UFP particles (upper panel) and ^99m^Tc-DTPA (lower panel) into perfusate of isolated perfused rat lung as a fraction of the deposited dose for inhaled particles and as fraction of the instilled dose for DTPA. Both control lungs (●) were used as well as lungs treated in-situ with H_2_O_2 _bolus 0.5 mM (O), histamine (1 μM) in perfusate (□) or 0,5 mM instilled into the lungs (■). A stabilisation period of 15 minutes is done before treatment and collection of samples are taken place every 15 minutes for translocation and lung markers detection. Values in the upper panel depict the mean and SD's of 3 or 4 experiments; values in the lower panel depict only the mean of 3 or 4 experiments indicating the trend.

**Figure 5:**
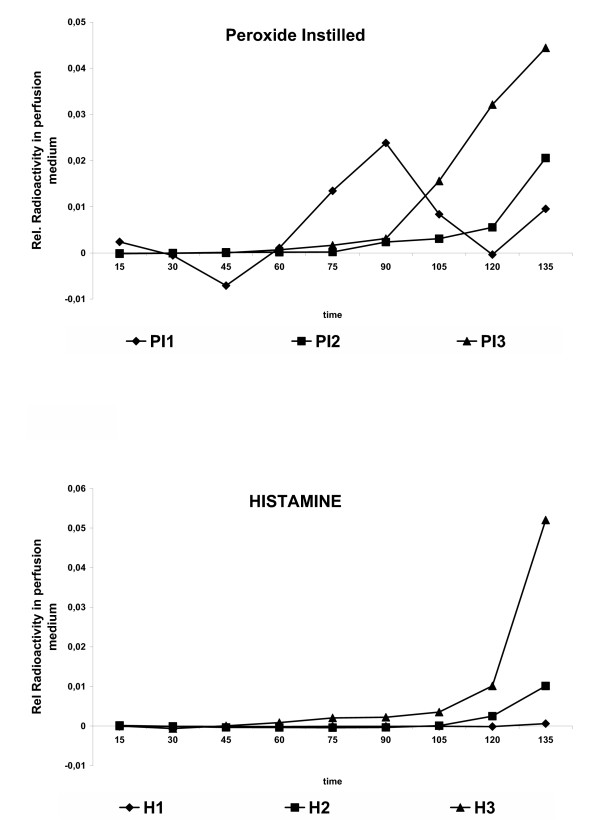
Translocation of Iridium particles in individual perfusions after treatment with (A) H_2_O_2 _(bolus) and (B) histamine (10 μM) in the perfusate. Translocation represented as relative radioactivity of ^192^Ir in the perfusate. Data shown for individual heart-lung-blocs.

In a large set of perfusions in control lungs no translocation of ^192^Ir-UFP particles was noted and the variance between different perfusions is small (< 5 %) as shown in Fig. 4A. Then several treatments were applied to investigate the role of epithelial and endothelial permeability on particle translocation. First, hyperinflation to double tidal volume every minute was applied but did not lead to increased translocation of nanoparticles (data not shown). An initial bolus injection of H_2_O_2 _into the trachea of the IPL to reach a final concentration of 0.5 mM, caused particle translocation to start at 60 min after onset of the inhalation of radioactive aerosol. A significant difference (P < 0.05, Mann-Whitney U-test) in particle-related radioactivity in the perfusate was observed between control and H_2_O_2 _group at 90, 105 and 120 minutes after onset of inhalation (Fig. 4A). At other time-points beyond 60 minutes the differences to untreated lungs were of borderline significance (P < 0,1; Mann Whitney-test). The variance between perfusions upon this treatment in Fig 4A was much higher than in control perfusions. However, individual presentation of the experiments of H_2_O_2 _pretreated lungs (Figure 5A) show a similar trend in all perfusions. Increased radioactivity in perfusate was only detected beyond 60 minutes of perfusion. A similar translocation versus time profile was observed in lungs upon presence of 1 μM histamine in the vascular perfusion fluid (Fig 4A). However, here statistical significance in this condition versus control lungs was only attained after 120 minutes of perfusion (Fig 4A), which is best explained by the individual experiments shown in Fig 5B. On the other hand, in the lungs treated with a histamine bolus injection no ^192^Ir-UFP radioactivity was detected in the perfusate.

Interestingly, the kinetics of translocation of DTPA (Fig. 4B) and Ir-UFP are very different. Whereas Ir-UFP only starts to increase in perfusate after 60 min of inhalation, DTPA is measured in perfusate within a few minutes after intratracheal instillation. On the other hand the effects of H_2_O_2 _and vascular histamine on particle translocation are also reflected in the DTPA -clearance (Fig. 4B). Although not significant, both treatments caused trends of a higher rate of translocation of DTPA one hour after administration, which is also observed for translocation of ^192^Ir-UFP. The histamine bolus injection, with a final target concentration in the lumen of 0.5 mM caused a considerable slowing-down of DTPA permeability (Fig 4B) and no observed effects on ^192^Ir-UFP translocation.

### Biomarkers of epithelial and endothelial damage

**Figure 6:**
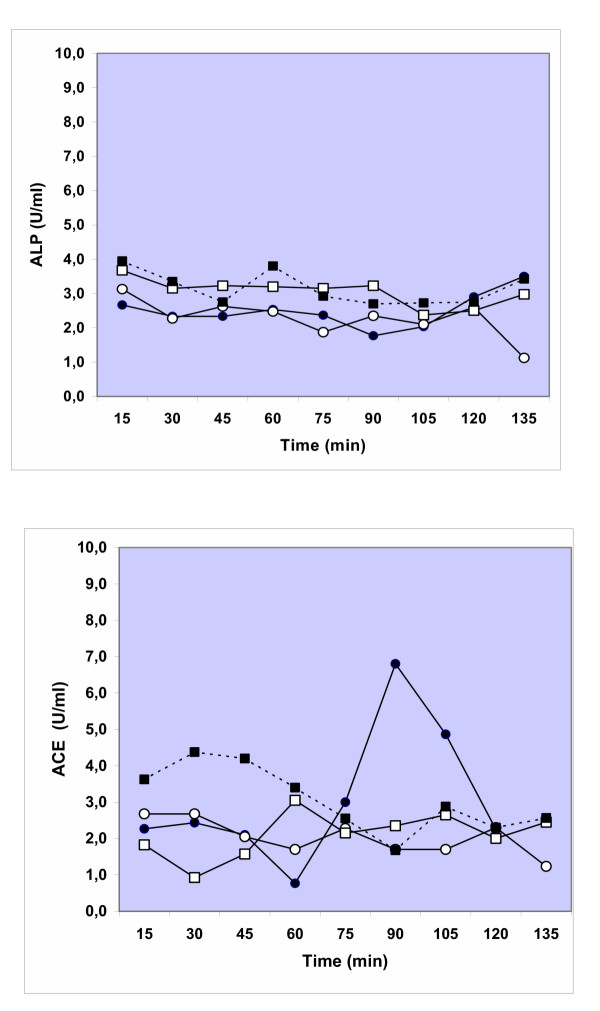
Release of Alkaline phosphatase (A) and Angiotensin converting enzyme (B) measured in lung perfusate during and after particle inhalation and different pre-treatments. Both control lungs (●) were used as well as lungs treated with H_2_O_2 _(O), histamine (10 μM) in perfusate (□) or injected into the lungs (■). Values depict the mean of 3 or 4 experiments.

Alkaline phosphatase (ALP) was measured in control and pre-treated lungs as a marker of type II cell damage. No significant differences in ALP activity (15–135 minutes) were observed between perfusate of the control IPL and H_2_O_2 _pre-treated lungs after exposure to ^192^Ir-UFP (Fig 6A). In the IPL perfused with vascular histamine a significantly lower activity of ALP was seen at 15 and 30 minutes in comparison to lung perfusions that only received ^192^Ir-UFP by inhalation. At all later time points ALP showed no difference to control lungs (Fig 6A). The histamine bolus group did also not differ from control group. To evaluate endothelial damage, angiotensin converting enzyme (ACE) was measured in the lung perfusate (Figure 6B). No significant differences were observed in ACE activity between the control group and isolated lungs treated with H_2_O_2_, histamine in perfusate or histamine delivered as a bolus in the trachea (ANOVA, post-hoc Tukey and Mann Whitney-test). To check whether the H_2_O_2 _effect was mediated by histamine release we measured histamine in the perfusate but did not detect significant differences to histamine levels in control perfusions (data not shown). Also a measure of total protein for lung permeability did not detect differences between the different treatments used in these experiments.

### Morphology of lungs

**Figure 7:**
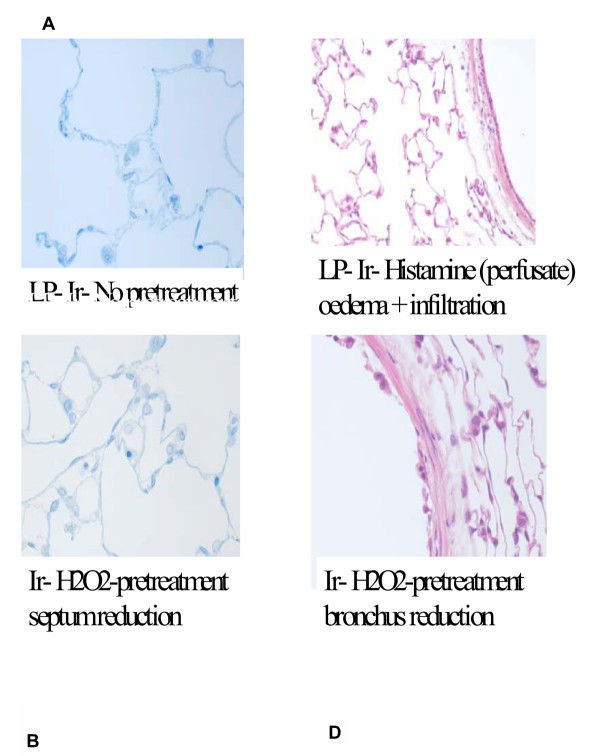
Examples of histopathological lesions encountered in lungs after 2 hour perfusion and inhalation of non-radioactive ^192^Ir-UFP in control lungs (A), lungs pretreated with a bolus of H2O2 in the lumen (B, D), showing septum and bronchus reduction, and (C) lung perfused with histamine, showing oedema and infiltration.

The outcomes of the histo-pathological analyses are summarised in Table 1 and illustrated in Fig. 7. Overall there is quite extensive damage at the end of the perfusion experiments, but there are not many differences between the different treatments. Sub-epithelial round cell infiltration, interstitial dilation along with moderate to severe oedema was present in all the groups. Occasionally alveolar dilation, alveolar inflammation and fluid in the alveolar lumen were detected. The in vitro perfusion procedure might be responsible for the perivascular and peribronchial dilation in all the lungs. H_2_O_2 _might be directly responsible for the epithelial damage of the proximal bronchi in the H_2_O_2 _instilled group.

**Table 1:** Morphological evaluation of the rat lungs after 2 hours perfusion and inhalation exposure to Ir-UFP, using lung sections and HE- or toluidine blue staining The regions investigated included the bronchial segment, the blood vessels and the alveolar region

**Treatment**	**Bronchial region**	**Blood vessels**	**Alveolar region**
**Control**	Sub epithelial round cell filtration; interstitial dilation	Interstitial dilation	Small alveolar dilation in periphery
**Hydrogen peroxide**	Desquamation of epithelial layer, partially destruction of sub epithelial structure	Moderate to severe dilation	
**Histamine Perfusate**	Sub epithelial round cell filtration; interstitial dilation	Moderate to severe interstitial dilation	Alveolar dilation in periphery
**Histamine (Bolus)**	Sub epithelial round cell filtration; interstitial dilation	Moderate to severe interstitial dilation	Focal alveolar dilation, oedematous fluid in alveolar lumen

## Discussion

The purpose of our study was to evaluate the role of epithelial and endothelial barrier in the translocation of ultrafine particles across the lung into the systemic circulation using the isolated perfused lung model. Translocation of Iridium (Ir)particles was monitored by radioactivity of the particles themselves, and not by any attached radioactive label. No translocation of ^192^Ir-UFP (17–20 nm) was detected in isolated perfused rat lungs. However lungs pre-treated *in-situ *with histamine on the endothelial side (1 μM) or H_2_O_2 _(0.5 mM) in the alveolar lumen showed small amounts of radioactivity in the single pass perfusate after a lag-time of 60 min. Although kinetics of DTPA and particle translocation were different the *in-situ *treatments histamine and H_2_O_2 _caused unidirectional in both processes, in the absence of biochemical evidence for epithelial and endothelial damage.

In this study we applied several *ex-vivo *treatments of the isolated lungs with either H_2_O_2 _or histamine. Using H_2_O_2 _we anticipated inducing an oxidative stress, which is also caused by PM inhalation in the lung both by direct radical formation by PM constituents and indirectly by recruited inflammatory cells (review: [34]). Oxidative stress has been forwarded as a central hypothetical mechanism in the adverse effects of PM, including ultrafine particles [35]. Actually, the oxidative capacity of PM was shown by us to be a predictor of bronchial inflammatory response to PM after installation in normal human volunteers [26]. Earlier on Rhaman et al [36] forwarded that oxidative stress and depletion of GSH can affect lung permeability allowing for greater particle passage via lung epithelium into the interstitium. This concept is supported by our data using a high concentration of H_2_O_2 _(5 mM) by bolus injection into the lung, to reach a final concentration of 0.5 mM. In fact, lung-lining fluid of COPD patients has been shown to contain levels of H_2_O_2 _up to 5 μM [37]. Although this model does certainly not meet all conditions of an inflammatory response, similar models have been applied in other ex-vivo permeability studies [38,39]. In isolated perfused rat lungs, a low concentration of H_2_O_2 _(0.25 mM) in the perfusate was shown to increase capillary permeability in the absence of lipid peroxidation [38]. A short-term treatment with H_2_O_2 _(100 mM) on the epithelium of human airway tubes caused a six-fold increase in translocation of ^111^In-DTPA, which was explained by the opening of paracellular pathways [39]. In our study, we assume that a final luminal concentration of 0.5 mM H_2_O_2 _is reached and we found an increased translocation of both ^192^Ir-UFP as well as a trend of increased translocation of ^99m^Tc-DTPA after a lag-time of about 60 min. However, no temporal relationship between both markers of translocation was seen, which suggest that they operate through different routes.

More data on the translocation route of ^192^Ir-UFP in the lungs are given by the results obtained with histamine administered both on the luminal side and through the microvasculature. These data show that histamine at very low levels in the perfusate (1 μM) caused an increased translocation of particles as well as ^99m^Tc-DTPA-permeation after a lag-time of about 60 min (Fig 4). On the other hand luminal administration of histamine (0.5 mM) did not increase ^192^Ir-UFP translocation and actually showed a slightly reduced ^99m^Tc-DTPA permeation. Histamine is a very potent vasoactive and bronchial mediator that has been shown to be involved in both local and systemic effects of diesel particles [40]. First, upon mast cell degranulation histamine is the major mediator of bronchial constriction as observed in allergic airway response [41]. This constriction probably also explains our reduction in ^99m^Tc-DTPA clearance after a histamine bolus injection into the trachea. The approach using vascular histamine is relevant because histamine has been shown to increase after instillation of particles in isolated tracheally perfused rabbit lung ex-vivo (Nemmar et al, 1999)[42], hamsters in vivo (Nemmar et al, 2003)[11] and healthy human volunteers (Salvi et al, 1999)[43]. We assume that histamine at low concentrations (10 ^-6 ^M) in our system increases endothelial permeability [44] and allows an increase in intercellular transport of ^192^Ir-UFP located in the interstitium through the endothelium into the perfusate. A similar effect of 10^-4 ^M vascular histamine was found recently in isolated perfused rabbit lungs (Nemmar et al, 2005)[45]. In the latter study latex particles (24–190 nm) translocated from the vascular compartment into the lumen, as observed by subsequent bronchoalveolar lavage. The amount of reverse translocation was about 2.5 % of administered dose within 2 hrs of perfusion. No translocation of latex particles with different size (24–190 nm) and surface chemistry (carboxylate versus amine) and charge were seen under normal physiological conditions in the rabbit lungs. In our studies 18 nm iridium particles also did not translocate through rat lung barriers, although we used negative pressure ventilation which caused considerable damage to the lung tissue.

Taken together these findings suggest that the translocation of ultrafine particles in the lung occur through different routes. Among different uptake routes we can discriminate transcytosis and para(inter)-cellular transport. Hermans et al [46] stated that radiolabelled tracers such as ^99m^Tc-labelled DTPA permeate the epithelial barrier by passing through intercellular junctions. Since kinetics of DTPA and ^192^Ir-UFP translocation in untreated lungs is so different, we suggest that ^192^Ir-UFP are translocated along different pathways. In fact, ultrafine particles may use different transcytotic pathways such as clathrin-coated pits, pinocytosis and non-coated pits, called caveolae [47]. Caveolae are the most likely route of uptake for the ^192^Ir-UFP used in our study (18 nm) as derived from studies by Gumbleton [47]. The alveolar epithelial cells, comprising 95 % of the lung surface have a cell thickness of 400 nm and about 600.000 to 900.000 caveolae that are 50–60 nm wide. Such a high number of possible transport units lead to assumption that NP smaller than 60 nm can be rapidly taken up and transported through the epithelial cell layer. Recently Kato et al (2003)[48] showed that lecithin-coated polystyrene latex beads (240 nm) got incorporated into the Type I and II alveolar epithelial cells as well as in the capillary lumen. It was suggested that these latex beads move from the alveolar epithelial cells to the capillary lumen via transcytosis. Kapp et al (2004)[49] found Ti0_2 _(29 nm) particles as intracellular clusters forming needle shape particles or rounded shape particles. This may also explain the relatively low and variable translocation as observed in other, previous *in vivo *studies [15-18]. We therefore used histamine to modify or facilitate trans-endothelial passage and indeed found that this enhanced translocation of ^192^Ir-UFP to the perfusate. However, we cannot discriminate between transcytosis and para-cellular transport in endothelium. We assume that the long lag-time (60 min) detected which is needed before passage is caused by the fact that an interstitial load has to be built by epithelial passage of ^192^Ir-UFP in the lung. The methods used in our study and the study by Nemmar et al (2005)[45] do not allow to evaluate whether translocation has occurred through primary particles or by aggregates. The inhalation in our study assured single UFP deposition in the alveolar region and virtually no agglomeration on the epithelium because of the alveolar surface and the number of deposited particles. However, upon vascular injection aggregates are formed, unless surface modifications are used that impede this process. It may therefore be that translocation observed in Nemmar's study occurs as aggregates by above mechanisms, or facilitation by phagocytic cells but not in our study. With this respect the studies by Heckel et al (2004)[50] have demonstrated by TEM that 4 nm gold-particles really pass membranes and reach the lumen as single particles. The difference between 4 and 20 nm particles may however be huge since 4 nm AU-particles are not recognized by the reticulo-endothelial system.

Our findings may be criticized due to a number of factors that are associated to our experimental design and performance. First, it must be taken into account that isolated and perfused lungs are not under physiological conditions since, for example, lymph flow is altered, bronchial perfusion is suppressed, autonomic innervation is disconnected and no blood cells (including inflammatory cells) are present in the perfusate. However the artificial negative pressure perfusion of isolated lung resembles respiratory conditions and the time span of experiments was limited up to 2 hours maximum to avoid excessive decrease of function. Knowing this, the lack of concomitant physiological measurement in the negative pressure ventilation is a major shortcoming in our data. The obvious reason for this is that given the use of radioactive ^192^Ir-UFP inclusion of measurement devices was allowed for radiation protection safety reasons, since they could lead to an open system and radioactive particle emissions. We have tried to compensate for this lack of know-how by measurement of biochemical indices of damage in perfusate and performing histology in lung after the experiment. No evidence for extensive lung damage in control conditions or after in-situ treatment to the essential barriers of the lung was noted. The release of ALP and ACE as biomarkers of epithelial and endothelial integrity are not elevated during the perfusion and do not correspond to the small translocation of ^192^Ir after a lag-time of about 60 min -UFP. Although microscopical analysis in lung sections showed desquamation of the epithelial layer in the lungs treated in situ with hydrogen peroxide, ALP levels in perfusate were not different from that in the control group. The experimental conditions also did not affect the integrity of the endothelial layer since perfusate levels of ACE did not change during perfusion. However, in most lungs oedema was noted in microscopy as interstitial dilation (Table 1) at the end of the experiment. The formation of oedema is due to the imbalance between fluid transvascular filtration and clearance. Also the excess fluid causes an overhydration of the interstitium associated with accumulation of oedema fluid in the loose connective tissue [51]. It could be argued that oedema might affect the translocation of ^192^Ir-UFP from the lumen via the interstitium across the endothelium. In fact, a recent study using iv injection of colloidal gold particles (4 nm) in rabbits, showed that a small but significant percentage (7 %) of the UFP was taken up in endothelial and epithelial cells of the lung [50]. After LPS infusion, causing mild pulmonary oedema, transendothelial transport was boosted five-fold, while a significant amount of gold particles accumulated in the interstitium (14 %) and even reached the alveoli (11 %). Although this suggests a potential interference for oedema in our study, one should realize that in the above study [50] oedema was present at the very beginning and translocation is followed in a different direction. Nevertheless an effect of oedema on particle translocation is considered unlikely in our experimental setup since no ^192^Ir-UFP translocation was observed in the control groups and after a histamine bolus for up to 2 hours after onset of inhalation.

The minute translocation of ^192^Ir-UFP in the isolated lung perfusion system conforms to our previous in vivo findings [15] using the same particles by inhalation at similar dose in rats. Other studies however reported very different amounts and kinetics of translocation. Nemmar et al [16] studied particle translocation after intratracheal instillation of uF particles in hamsters *in vivo *and observed a rapid (3 % within 5 min) translocation of 80 nm albumin particles coated with ^99m^Tc but no translocation was observed after the 15 minutes. On the other hand, the same group could not find latex (24–190 nm) particle translocation isolated perfused rabbit lungs at positive pressure [45]. Surface chemistry did not affect this process. In a similar approach using latex fluorescent beads and positive pressure ventilation, we also did not find particle translocation (data not shown). In contrast Brooking et al [48] showed a continuous increase in translocation with time up to 180 minutes during nasal inhalation of latex particles between 50 and 250 nm by rats. The latter study highlighted the importance of particle size as the smallest ultrafine particles showed higher uptake rates than the larger particles. In addition they demonstrated that particle surface chemistry was an important characteristic [52]. Oberdorster et al [18] reported quite extensive (> 20 %) translocation of uF carbon particles (18 nm) after short-term inhalation. Whatever the mechanism or particle properties involved in passing the lung barriers, the question remains what particle translocation means in terms of systemic effects. The mainstream hypothesis is that lung inflammation causes and facilitates the release of mediators that adversely affect cardiovascular parameters [3]. Alternatively, translocation of particles to the brain (Oberdorster et al, 2004)[53] or the systemic circulation may also explain effects of PM exposure on heart and vascular tissue. The blood that leaves the lung first enters the heart before it is pumped to the other organs. In our previous work we showed that suspensions or filtrates of PM_10 _could have direct effects on vessels [9]. However, the effects were rather due to the soluble components (i.e. transition metals) than to particles themselves [9] and are in contrast to *in vivo *findings on endothelial function with PM [54,10] 2004).

Although these data do not allow quantitative conclusions on the exact mechanism and the importance of systemic translocation of UFP as a mechanism in adverse effects of PM, we do confirm that ultrafine particles can translocate from the lung into the circulation using the isolated perfused rat lung upon pharmacological mediation. Permeability of the lung barrier to ultrafine particles seems to be controlled both at the epithelial and endothelial level and conditions that affect this barrier function such as inflammation may affect translocation of UFP. The conditions under which this does occur mimic conditions that are met in diseased, susceptible subjects including asthmatics and COPD-patients.

## References

[B1] Dockery DW, Pope CA, Xu X, Spengler JD, Ware JH, Fay ME, Ferris BG, Speizer FE (1993). An association between air pollution and mortality in six U.S. cities. N Engl J Med.

[B2] Pope CA, Burnett RT, Thun MJ, Calle EE, Krewski D, Ito K, Thurston GD (2002). Lung cancer, cardiopulmonary mortality, and long-term exposure to fine particulate air pollution. JAMA.

[B3] Seaton A, MacNee W, Donaldson K, Godden D (1995). Particulate air pollution and acute health effects. Lancet.

[B4] Hoek G, Dockery DW, Pope A, Neas L, Roemer W, Brunekreef B (1998). Association between PM10 and decrements in peak expiratory flow rates in children: reanalysis of data from five panel studies. Eur Respir J.

[B5] Peters A, Doring A, Wichmann HE, Koenig W (1997). Increased plasma viscosity during an air pollution episode: a link to mortality?. Lancet.

[B6] Gold DR, Litonjua A, Schwartz J, Lovett E, Larson A, Nearing B, Allen G, Verrier M, Cherry R, Verrier R (2000). Ambient pollution and heart rate variability. Circulation.

[B7] Pope CA, Verrier RL, Lovett EG, Larson AC, Raizenne ME, Kanner RE, Schwartz J, Villegas GM, Gold DR, Dockery DW (1999). Heart rate variability associated with particulate air pollution. Am Heart J.

[B8] Suwa T, Hogg JC, Quinlan KB, Ohgami A, Vincent R, van Eeden SF (2002). Particulate air pollution induces progression of atherosclerosis. J Am Coll Cardiol.

[B9] Bagate K, Meiring JJ, Gerlofs-Nijland, Vincent R, Cassee F, Borm PJA (2004). Vascular effects of particle instillation in spontaneous hypertensive rats. Toxicol Appl Pharmacol.

[B10] Nurkiewicz TR, Porter DW, Barger M, Castranova V, Boegehold MA (2004). Particulate matter exposure impairs systemic microvascular endothelium-dependent dilation. Environ Health Perspect.

[B11] Nemmar A, Hoylaerts MF, Hoet PH, Dinsdale D, Smith T, Xu H, Vermylen J, Nemery B (2002). Ultrafine particles affect experimental thrombosis in an in vivo hamster model. Am J Respir Crit Care Med.

[B12] Li N, Sioutas C, Cho A, Schmitz D, Misra C, Sempf J, Wang M, Oberley T, Froines J, Nel A (2003). Ultrafine particulate pollutants induce oxidative stress and mitochondrial damage. Environ Health Perspect.

[B13] Stone V, Tuinman M, Vamvakopoulos JE, Shaw J, Brown D, Petterson S, Faux SP, Borm P, MacNee W, Michaelangeli F, Donaldson K (2000). Increased calcium influx in a monocytic cell line on exposure to ultrafine carbon black. Eur Respir J.

[B14] Oortgiesen M, Veronesi B, Eichenbaum G, Kiser PF, Simon SA (2000). Residual oil fly ash and charged polymers activate epithelial cells and nociceptive sensory neurons. Am J Physiol Lung Cell Mol Physiol.

[B15] Kreyling WG, Semmler M, Erbe F, Mayer P, Takenaka S, Schulz H, Oberdorster G, Ziesenis A (2002). Translocation of ultrafine insoluble iridium particles from lung epithelium to extrapulmonary organs is size dependent but very low. J Toxicol Environ Health A.

[B16] Nemmar A, Vanbilloen H, Hoylaerts MF, Hoet PH, Verbruggen A, Nemery B (2001). Passage of intratracheally instilled ultrafine particles from the lung into the systemic circulation in hamster. Am J Respir Crit Care Med.

[B17] Nemmar A, Hoet PH, Vanquickenborne B, Dinsdale D, Thomeer M, Hoylaerts MF, Vanbilloen H, Mortelmans L, Nemery B (2002). Passage of inhaled particles into the blood circulation in humans. Circulation.

[B18] Oberdorster G, Sharp Z, Atudorei V, Elder A, Gelein R, Lunts A, Kreyling W, Cox C (2002). Extrapulmonary translocation of ultrafine carbon particles following whole-body inhalation exposure of rats. J Toxicol Environ Health A.

[B19] Ferin J, Oberdorster G, Penney DP (1992). Pulmonary retention of ultrafine and fine particles in rats. Am J Respir Cell Mol Biol.

[B20] Borm PJ, Schins RP, Albrecht C (2004). Inhaled particles and lung cancer, part B: paradigms and risk assessment. Int J Cancer.

[B21] Bernard A, Hermanus C, Van Houte G (1997). Transient increase of serum Clara cell protein (CC16) after exposure to smoke. Occup Environ Med.

[B22] Royston BD, Webster NR, Nunn JF (1990). Time course of changes in lung permeability and edema in the rat exposed to 100 % oxygen. J Appl Physiol.

[B23] Hamoir J, Nemmar A, Halloy D, Wirth D, Vincke G, Vanderplasschen A, Nemery B, Gustin P (2003). Effect of polystyrene particles on lung microvascular permeability in isolated perfused rabbit lungs: role of size and surface properties. Toxicol Appl Pharmacol.

[B24] Nemmar A, Hoet PH, Vermylen J, Nemery B, Hoylaerts MF (2004). Pharmacological stabilization of mast cells abrogates late thrombotic events induced by diesel exhaust particles in hamsters. Circulation.

[B25] Abbott NJ (2000). Inflammatory mediators and modulation of blood-brain barrier permeability. Cell Mol Neurobiol.

[B26] Schaumann F, Borm PJ, Herbrich A, Knoch J, Pitz M, Schins RP, Luettig B, Hohlfeld JM, Heinrich J, Krug N (2004). Metal-rich Ambient Particles (PM2.5) Cause Airway Inflammation in Healthy Subjects. Am J Respir Crit Care Med.

[B27] Dick CA, Singh P, Daniels M, Evansky P, Becker S, Gilmour MI Murine pulmonary inflammatory responses following instillation of size-fractionated ambient particulate matter. J Toxicol Environ Health A.

[B28] Mullin JM, Snock KV (1990). Effect of Tumor necrosis factor on epithelial tight junctions and transepithelial permeability. Cancer Research.

[B29] Lee YM, Hybertson BM, Cho HG, Terada LS, Cho O, Repine AJ, Repine JE (2000). Platelet-activating factor contributes to acute lung leak in rats given interleukin-1 intratracheally. Am J Physiol Lung Cell Mol Physiol.

[B30] Uhlig S, Wollin L (1994). An improved setup for the isolated perfused rat lung. :. J Pharmacol Toxicol Methods.

[B31] Maguire GA, Price CP (1985). A continuous monitoring spectrophotometric method for the measurement of angiotensin-converting enzyme in human serum. Ann Clin Biochem.

[B32] Smith PK, Krohn RI, Hermanson GT, Mallia AK, Gartner FH, Provenzano MD, Fujimoto EK, Goeke NM, Olson BJ, Klenk DC (1985). Measurement of protein using icinchoninic acid. Anal Biochem.

[B33] Fischbach F, Zawta B (1992). Age-dependent reference limits of several enzymes in plasma at different measuring temperatures. Klin Lab.

[B34] Knaapen AM, Borm PJA, Albrecht C, Schins RPF (2004). Inhaled particles and Lung cancer. Part A: Mechanisms. Int J Cancer.

[B35] Donaldson K, Stone V, Borm PJ, Jimenez LA, Gilmour PS, Schins RP, Knaapen AM, Rahman I, Faux SP, Brown DM, MacNee W Oxidative stress and calcium signaling in the adverse effects of environmental particles (PM10). Free Radic Biol Med.

[B36] Rahman I, Mulier B, Gilmour PS, Watchorn T, Donaldson K, Jeffery PK, MacNee W Oxidant-mediated lung epithelial cell tolerance: the role of intracellular glutathione and nuclear factor-kappaB. Biochem Pharmacol.

[B37] De Benedetto F, Aceto A, Dragani B, Spacone A, Formisano S, Cocco R, Sanguinetti CM (2000). Validation of a new technique to assess exhaled hydrogen peroxide: results from normals and COPD patients. Monaldi Arch Chest Dis.

[B38] Habib MP, Clements NC (1995). Effects of low-dose hydrogen peroxide in the isolated perfused rat lung. Exp Lung Res.

[B39] Hulsmann AR, Raatgeep HR, den Hollander JC, Bakker WH, Saxena PR, de Jongste JC (1996). Permeability of human isolated airways increases after hydrogen peroxide and poly-L-arginine. Am J Respir Crit Care Med.

[B40] Nemmar A, Nemery B, Hoet PH, Vermylen J, Hoylaerts MF Pulmonary inflammation and thrombogenicity caused by diesel particles in hamsters: role of histamine. Am J Respir Crit Care Med.

[B41] Nadel JA, Barnes PJ (1984). Autonomic regulation of the airways. Annu Rev Med.

[B42] Nemmar A, Delaunois A, Nemery B, Dessy-Doize C, Beckers JF, Sulon J, Gustin P Inflammatory effect of intratracheal instillation of ultrafine particles in the rabbit: role of C-fiber and mast cells. Toxicol Appl Pharmacol.

[B43] Salvi S, Blomberg A, Rudell B, Kelly F, Sandstrom T, Holgate ST, Frew A (1999). Acute inflammatory responses in the airways and peripheral blood after short-term exposure to diesel exhaust in healthy human volunteers. Am J Respir Crit Care Med.

[B44] Leach L, Eaton BM, Westcott ED, Firth JA (1995). Effect of histamine on endothelial permeability and structure and adhesion molecules of the paracellular junctions of perfused human placental microvessels. Microvasc Res.

[B45] Nemmar A, Hamoir J, Nemery B, Gustin P Evaluation of particle translocation across the alveolo-capillary barrier in isolated perfused rabbit lung model. Toxicology.

[B46] Hermans C, Bernard A (1999). Lung epithelium-specific proteins: characteristics and potential applications as markers. Am J Respir Crit Care Med.

[B47] Gumbleton M Caveolae as potential macromolecule trafficking compartments within alveolar epithelium. Adv Drug Deliv Rev.

[B48] Kato T, Yashiro T, Murata Y, Herbert DC, Oshikawa K, Bando M, Ohno S, Sugiyama Y (2003). Evidence that exogenous substances can be phagocytized by alveolar epithelial cells and transported into blood capillaries. Cell Tissue Res.

[B49] Kapp N, Kreyling W, Schulz H, Im Hof V, Gehr P, Semmler M, Geiser M Electron energy loss spectroscopy for analysis of inhaled ultrafine particles in rat lungs. Microsc Res Tech.

[B50] Heckel K, Kiefmann R, Dorger M, Stoeckelhuber M, Goetz AE (2004). Colloidal gold particles as a new in vivo marker of early acute lung injury. Am J Physiol Lung Cell Mol Physiol.

[B51] Bayat S, Grimbert F, Uhlig S, Taylor AE (1998). Experimental and clinical measurement of pulmonary edema.

[B52] Brooking J, Davis SS, Illum L (2001). Transport of nanoparticles across the rat nasal mucosa. J Drug Target.

[B53] Oberdorster G, Sharp Z, Atudorei V, Elder A, Gelein R, Kreyling W, Cox C (2004). Translocation of inhaled ultrafine particles to the brain. Inhal Toxicol.

[B54] Brook RD, Brook JR, Urch B, Vincent R, Rajagopalan S, Silverman F Inhalation of fine particulate air pollution and ozone causes acute arterial vasoconstriction in healthy adults. Circulation.

